# Photodissociation-driven photoacoustic spectroscopy with UV-LEDs for ozone detection

**DOI:** 10.1016/j.pacs.2025.100718

**Published:** 2025-03-29

**Authors:** Lukas Escher, Thomas Rück, Simon Jobst, Jonas Pangerl, Rudolf Bierl, Frank-Michael Matysik

**Affiliations:** a Sensorik-ApplikationsZentrum (SappZ) der Ostbayerischen Technischen Hochschule (OTH) Regensburg, Regensburg 93053, Germany; b Institut für Analytische Chemie, Chemo, und Biosensorik, Universität Regensburg, Regensburg 93053, Germany

**Keywords:** Photoacoustic spectroscopy, UV-LED, Ozone, Photodissociation, Cross-sensitivity

## Abstract

This study presents the development and evaluation of a UV-LED based photoacoustic (PA) measurement system for ozone (O_3_) detection to demonstrate its potential for low-cost and accurate sensing while for the first time addressing the importance of photodissociation for PA signal generation for O_3_ in the UV range. With a detection limit of 7.9 ppbV, the system exhibits a significant advancement over state-of-the-art UV-PA O_3_ detection and is on par with laser-based setups. Following a novel discussion of the PA signal arising from photodissociation and its products, cross-sensitivity effects due to environmental factors such as temperature and gas composition were systematically analyzed. A digital twin driven compensation for these influences was implemented and evaluated. Despite the challenges associated with modeling the effects of H_2_O and CO_2_, the PA system shows considerable potential, though further studies in real world applications must be conducted.

## 1 Introduction

The ozone layer in the stratosphere has been extensively studied within the topic of atmospheric research [1], [2], [3]. However, the investigation of ozone concentrations at ground level, formed through photochemical reactions [4] is also of serious interest as elevated ozone concentrations have been found to adversely affect both the environment and public health [5], [6], [7], [8]. Therefore, the established air quality guidelines recommend a maximum daily 8 h average exposure of 100 µg/m^3^ (equivalent to 51 ppbV at 25°C and 1013 hPa) [5]. Furthermore, ozone pollution causes damage to vegetation [9], [10], potentially reducing yields and growth via various interactions with plants and pollinators [11]. Given the intensive impacts on the environment, it is essential to monitor ground level ozone concentrations to verify the effectiveness of emission-reducing measures.

In addition, an increasing trend in the number of indoor applications that are based on higher ozone concentrations (>1 ppmV) has been observed [12]. Those include the disinfection and purification of water [13], of air in rooms or cars [14], and the utilization in food industry [15], [16]. The quantity of ozone released into a confined space during such operations may easily exceed public health standards, compromising indoor air quality. Consequently, it is necessary to apply systems that are capable of measuring O_3_ concentration over a larger dynamic range indoors [17].

While the human olfactory sense is capable of perceiving ozone in the ppbV range [18], prolonged exposure to the gas has been demonstrated to impair olfactory function and consequently the loss of the ability to detect the scent of ozone [19]. A more quantitative and reliable set of techniques that can be employed for ppbV level detection includes, for example, chemiluminescence detectors [20], electrochemical sensors [21] and the most commonly used direct absorption-based photometers [22], [23].

The technique of photoacoustic spectroscopy (PAS) has been established as a reliable method for the measurement of trace gases in scientific research due to its exceptional sensitivity and dynamic measurement range. In the context of O_3_ detection, a range of PAS approaches exploiting the gas infrared absorption features using either quantum cascade or CO_2_ lasers have been reported and evaluated in the laboratory achieving single digit ppbV levels of detection [24], [25], [26]. A CO_2_ laser-based instrument has also been used in a field test to monitor ground level O_3_ concentrations, where the PA device was coupled with a potassium hydroxide scrubber to mitigate the effect of bulk gas changes [27]. Further research utilizes lasers with emission wavelengths within the Chappuis absorption band in the visible range in conjunction with PAS. Köhring et al. achieved a limit of detection (LOD; 1σ) of 2.13 ppmV employing quartz enhanced PAS (QEPAS) and a frequency doubled Nd:YAG laser in conjunction with a direct absorption reference device [28]. Fischer et al. and Cotterell et al. both report the use of O_3_ as calibration for aerosol photoacoustic spectrometers [29], [30]. By utilizing multiple laser diodes spanning the Chappuis band and referencing against cavity ringdown spectrometers (CRDS), they also studied photodissociation in the visible range and observed and modeled PA cross-sensitivities towards O_2_ concentration. Most recently, Keeratirawee and Hauser reported on microphone-based PAS detection of O_3_ employing a red laser, mentioning a LOD of 1.6 ppmV, though no in-line O_3_ measurement device was employed for reference, which is required for thorough investigations in the context of O_3_ detection [31]. Besides, no account was taken for flow or bulk-specific interferences.

However, the strongest absorption cross-section of O_3_ is the Hartley band, which is located in the UV range. PAS has already been investigated in the UV region without using a reference device, by employing microphones and expensive frequency-quadrupled Nd:YAG UV lasers, achieving a LOD of 2.8 ppbV [32] and 10 ppbV [33], respectively. Utilizing more affordable but low power UV-LEDs in combination with QEPAS, Böttger et al. reported a detection limit of 1.27 ppmV referenced against a direct absorption monitor [34]. However, literature does not yet address either PA signal generation or cross-sensitivities following photodissociation in the Hartley band, which forms the basis of this work.

Nonetheless, recent advancements in the field of UV-LED technology have led to a further reduction in cost and an increased output power of these devices. The objective of this research is to utilize this development to establish a cost-effective PAS measurement setup based on a UV-LED that achieves a sufficiently low limit of detection in order to provide reliable performance. Accompanying this, our goal is mainly to conduct an analysis of the PA signal origin regarding UV-based O_3_ detection. This first step is imperative to facilitate a comprehensive assessment of the PAS relevant cross-sensitivities.

## 2 Methods

### 2.1 Photoacoustic spectroscopy

PAS is a well-established trace gas detection method the theory of which has been often described in detail [35], [36]. The amplitude-modulated photoacoustic (PA) signal is demodulated by a lock-in amplifier (LIA) for signal-to-noise ratio (SNR) enhancement, yielding a voltage signal $U_{\text{LIA}}$ [36],

(1) $$U_{\text{LIA}}=\frac{1}{\sqrt{2}}U_{\text{mic}}=C_{\text{corr}}B_{\text{mic}}\left(\gamma -1\right){\left(\frac{Q}{\omega }\right)}_{\text{res}}\frac{1}{\pi {\text{r}}^{2}}\frac{{\text{N}}_{\text{A}}}{V_{\text{mol}}}N_{\text{i}}\sigma_{\text{i}}\left(\lambda \right)P_{0}\epsilon_{\text{relax}}$$

**Fig. 1:**
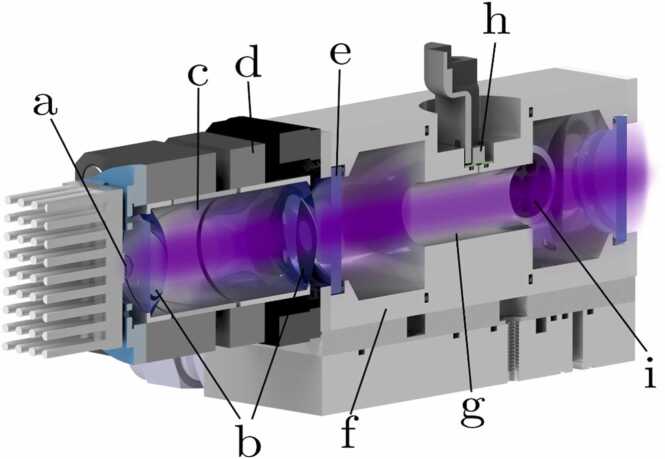
Half section of a rendered drawing of the aluminum PAC in combination with a UV-LED and its optic module: a) UV-LED covered by a half-dome lens on a PCB mounted on a heat sink, b) AR coated optics (aspheric and biconvex lens), c) polished aluminum reflectors covering the inside of the optics mounts, d) 3D printed optic mounts with magnetic flange to PAC, e) AR coated windows, f) temperature controlled aluminum PAC body with two buffer volumes and g) polished acoustic resonator, h) microphone with 3D printed mount, i) speaker for ARMS.

Here, the microphone voltage $U_{\text{mic}}$ depends on a number of parameters potentially influencing the signal in the event of changes in bulk gas composition or ambient parameters. When examining the influence of acoustic effects on the PA signal, it is essential to take into account the heat capacity ratio γ of the sample gas, the quality factor Q and the modulation frequency ω of the exploited acoustic resonance. This was achieved using an acoustic resonance monitoring system (ARMS) with a speaker integrated into the buffer volume of the PAC (visualized in Fig. 1; for a comprehensive description of the ARMS refer to [36]). Furthermore, Eq. (1) also regards the physical dimensions of the resonator tube of the PAC, such as the radius r, the Avogadro constant $N_{\text{A}}$, the molar volume of the sample $V_{\text{mol}}$ and the analytes dimensionless volume fraction $N_{\text{i}}$. It also includes the corresponding absorption cross-section $\sigma_{\text{i}}$ at a wavelength λ of the light source, which emits light with an optical power $P_{0}$. The pressure is converted to a voltage signal via the microphone with its sensitivity $B_{\text{mic}}$ and an empirically validated refinement factor $C_{\text{corr}}$, which represents setup-specific inaccuracies [36]. Finally, the efficiency of the total non-radiative relaxation $\epsilon_{\text{relax}}$ of the excited molecule must be considered. A change in bulk gas composition or environmental conditions might affect both the acoustic parameters and also the pathway of relaxation [35]. Readers seeking a more comprehensive derivation of the PA pressure are referred to reference [37].

When a new analyte is investigated for potential use in PAS, the relevant cross-sensitivities for the intended application are typically analyzed in a laboratory setting. With the exception of $\epsilon_{\text{relax}}$, each parameter in Eq. (1) can be measured or calculated analytically. If a measurement curve is analyzed for changes in ϵ, the equation is solved for ϵ, i.e. the measured voltage is compensated for all the other variables. For purely acoustic phenomena, a horizontal straight line can be expected when plotting ϵ against the varied parameter, since deviations in the observed signal can be entirely explained by the other variables (Q, ω, γ, $V_{\text{mol}}$). However, if a deviating, non-constant curve results, this indicates either a change in non-radiative relaxation efficiency during the analyzed measurement or other competing phenomena, such as, e.g. photodissociation or chemical reactions of components of the gas sample, which cannot be distinguished from a change in ϵ. This method was employed in the present study to analyze the conducted measurements for non-radiative relaxation or competing processes and to quantitatively isolate these from other known phenomena. In the case of non-radiative relaxation, a relaxation model can be established by researching collisional and quenching reactions and their rate constants in the available literature. Thus, their effect on the PA signal is compensated using the CoNRad algorithm [35] and digital twin (DT) [36] developed in previous works of our group.

### 2.2 UV-LED based PAS setup

The strong absorption of the Hartley band of O_3_ in the ultraviolet (UV) range [38] already permits highly precise measurements through direct absorption spectroscopy (DAS) [23]. Combining this with the recent development in higher power UV light emitting diodes (LEDs) [39], which are readily modulated electrically, enables precise O_3_ trace measurements with PAS as well.

Fig. 1 illustrates the half-section rendering of the PAC and the UV-LED module with collimation optics. The overall design is based on the preliminary work presented in reference [40]. In this work, the standard H-shaped metal 3D-printed PAC [37], [41] was transferred to a conventionally fabricated modular design approach. This enables easier customized production in larger quantities while retaining modularity and the same internal dimensions. The optic module is secured to the PAC by two magnets and comprises a UV-LED (LB6868-UCV-275 nm-D4, Ivy Bridge Technology (IBT), China) mounted on a printed circuit board (PCB) with a heat sink. A half-dome lens is positioned directly atop the chip. An aspherical lens (No. 33955, Edmund Optics, US) and a biconvex lens (No. 46290, Edmund Optics, US), both antireflective (AR) coated, direct the emitted light towards the PAC’s acoustic resonator (for more details refer to [40], [42]). The addition of polished aluminum reflector rings on the interior of the optic mounts further increases the optical power guided towards the PAC.

The aluminum PAC itself is temperature-controlled at 30 °C by a Peltier and NTC control loop (NTC: B57861S, TDK Electronics, Germany; Peltier: ET-161–12–08-E, European Thermodynamics Ltd., UK; TEC: TEC-1122-SV, Meerstetter Engineering GmbH, Switzerland) and the sample flow is monitored by a BME 280 temperature, pressure, and humidity (T,p,H) sensor (Bosch Sensoric GmbH, Germany; these components are not shown in the figure for the sake of clarity). To ensure gas-tightness of the PAC and, consequently, the integrity of the supplied calibration gases, two AR coated windows (No. 65875, Edmund Optics, US) seal the cell. The polished acoustic resonator tube in the center of the cell, with a diameter of 13 mm and length of 31 mm, is flanked by two buffer volumes (30 mm diameter and 19 mm length) which act as acoustic filters to reduce noise from the gas flow and to decouple the background signal from the windows. In the rear buffer volume, a speaker (K16, Visaton GmbH, Germany) is inserted to perform ARMS measurements. As a low noise sound transducer for measuring the standing wave that originates from the PA effect within the acoustic resonator, a micro-electromechanical system (MEMS) microphone (ICS-40730, TDK InvenSense, Germany) is positioned at the center of the resonator, atop a 1 mm decoupling hole.

The 275 nm LED is driven by a current driver (LED-Pulse-Controller 2K10, Leistungselektronik JENA GmbH, Germany) up to a maximum of 700 mA, with rectangular modulation provided by a function generator (33500 B, Keysight Technologies, US). The waste heat is transferred to a heat sink allocated directly on the LED circuit board, which is cooled by a fan. The optical power transmitted through the PAC is monitored by a thermal detector head (S302C detector and PM100D power meter console, Thorlabs GmbH, Germany). However, it should be noted, that due to the relatively smaller diameter of the sensor compared to the LED beam behind the PAC, only the central portion of the light is measured. Given that the signal frequency is determined by the modulation frequency of the LED, a lock-in amplifier (7270 General Purpose DSP Lock-in Amplifier, Signal Recovery/Ametek, US) is used, drastically enhancing the SNR. All devices utilized for controlling, reading out, and monitoring the PA setup are integrated in software, controlled and synchronized via a PC.

### 2.3 Experimental setup for ozone gas measurements

**Fig. 2:**
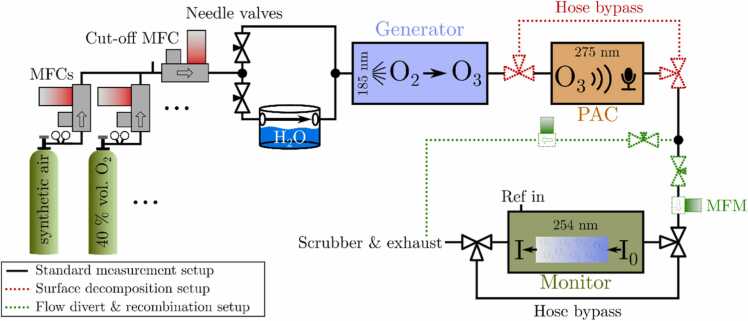
Block diagram illustrating the measurement and gas setup with its variations employed in this work. The sample gases are composed of premixed cylinders, i.e. synthetic air, N_2_, 40 % vol. O_2_ in N_2_ and 20 % vol. CO_2_ in synthetic air and in N_2_, respectively. After mixing, the flow can be humidified and is then passed through the O_3_ generator to the PAC. While the PAC can be bypassed (red dotted), the flow is subsequently divided once more after exiting the cell, as indicated by the two green dotted paths. The gas is finally analyzed by the O_3_ monitor, and afterwards scrubbed of O_3,_ and discharged. The dotted variations of the setup are only employed for the specified experiments and are otherwise omitted.

Fig. 2 provides an overview of the gas mixing setup and the configuration of the individual components within the system. The premixed pressurized gas containers are combined by mass flow controllers (MFCs), from which the flow can be diverted by two needle valves into a dry and a humid part. This allows for defined humidification of the sample. Subsequently, the gas enters the O_3_ generator, followed by the PAC. The PAC can be bypassed using PTFE (polytetrafluorethylene) tubing with two three-way valves (red dotted components), allowing for determination of the effect of the PAC’s surface on the O_3_ concentration by comparing the O_3_ concentration through both paths at a constant generator concentration. After the PAC, the flow can be divided once more with two needle valves to investigate the recombination effect of the photodissociation products exiting the PAC, which originate from the UV-LED light interacting with O_3_ (green dotted components; refer to Section 3.1 for further details). Both paths are monitored by a mass flow meter (MFM), the needle valves allow for finely adjusting the flow velocity and thus the residence time required for the gas to travel from the PAC to the O_3_ monitor. This method enables verification of the recombination process of the photodissociation products, giving insight and confidence into the origin of the PAS signal. Additionally, the O_3_ monitor can be bypassed by two three-way valves. This is used to prevent interfering sound originating from its switching solenoid valves to affect the PAS signal acoustically during PA measurement, i.e. the monitor is used for measuring the settling of the gas. Once this is ensured, reference data is recorded over a defined period. Then, the monitor is bypassed to allow undisturbed measurements with the PAC. The exhaust gas is cleaned by an O_3_ scrubber prior to discharge. It should be noted that the dotted lines representing measurement setup variations are only employed in the system during the specific measurements. Otherwise, only the solid black line marked standard measurement setup is used to maintain simplicity during all other measurements.

#### 2.3.1 Generating ozone sample gas

It has been demonstrated that ozone does not maintain a constant concentration when stored in a gas container at a higher concentration under non-humid conditions with synthetic air as buffer gas at room temperature [43], [44]. Therefore, to provide ozone samples an O_3_ generator (Ambient Ozone Simulator AOS 2, BMT Messtechnik GmbH, Germany) was utilized exploiting the O_2_ photodissociation induced by 185 nm radiation [45], [46] with a quantum yield of Φ=1 [47]

(2) O_2_+*hv*(185 nm)→O(^3^P)+O(^3^P)

and further the reaction of O(^3^P) with O_2_ to produce O_3_

(3) O(^3^P)+O_2_→O_3_+M

The desired O_3_ concentration can be adjusted by means of a potentiometer (Poti) at the AOS, which regulates the low-pressure mercury-vapor light intensity, reaching up to 6 ppmV O_3_ using synthetic air and 500 sccm flow (refer to appendix Fig. 9). Changing the oxygen concentration, or the flow rate of the sample gas also exerts a significant influence on the ozone concentration generated (correlation shown in appendix Fig. 10, Fig. 11), which, however, can distort the findings of cross-sensitivity measurements. Further, when examining the impact of humidity on the PAS signal, which is a prevalent parameter when measuring in air, it is essential to consider the influence of humidity on the generation of O_3_. H_2_O also absorbs the 185 nm radiation used for O_3_ generation and dissociates resulting in an OH product, which can subsequently react with O_3_ to effectively reduce the concentration of generated O_3_ (see Fig. 12 in the appendix) [46], [48], [49].

Furthermore, it is necessary to consider the effect of the gas system tubing and the PAC surface materials on the O_3_ concentration. Ozone is known to decompose on a lot of surfaces via a heterogeneous reaction, influenced by chemical and physical processes [50]. Metal surfaces in particular amplify this effect [43], leading to long settling times for stable O_3_ concentrations during steady flow settings (visualized exemplarily in appendix Fig. 13) [51]. Accordingly, the tubing from the outlet of the AOS in downstream direction is kept as short as possible and made of PTFE, as this showed less effects on the actual O_3_ concentration. Consequently, the only metal surface component remaining in the gas system that is exposed to O_3_ is the PAC made of aluminum. It is possible to passivate alumina surfaces by repeatedly exposing them to higher O_3_ concentrations for longer times [52], as observed in this work when frequently using the gas setup. This reduces the surface effect [51], particularly the time required for settling to a constant concentration to approximately 1 hour (see appendix Fig. 13). Though, reproducibility of the settling time is not always consistent, as it also depends on the time elapsed since the last O_3_ measurement. Using stainless steel PACs instead of aluminum yielded comparable results, with no discernible advantage of the heavier steel. Consequently, the aluminum variant was employed for all the experiments. However, the conditioning observed for the metal surfaces will inevitably deteriorate gradually in the absence of regular ozone exposure of the gas setup, which agrees with literature [51].

#### 2.3.2 Reference ozone monitor analysis

Given the necessity of considering all the effects described above in order to properly study the PA method, it is essential to incorporate a reference device for ozone detection into the gas system. Therefore, a device based on direct absorption (106-LFT, 2B Technologies, US) was employed as O_3_ reference. This device alternates every two seconds by switching of a solenoid valve between measuring the absorption of the sample gas (light intensity I) and the background signal drawn in through an ozone scrubber (light intensity I_0_) at the 254 nm line of a low-pressure mercury lamp, with the objective of determining the concentration according to Beer-Lambert law. The device was integrated as closely as possible downstream of the PAC. However, regarding the automated change between sample and reference, facilitated by electrically switching three-way valves, an acoustic interference is introduced into the PAC upstream due to the loud and abrupt sound of the switching valves. Therefore, the monitor was implemented with the option of bypassing it during PAS measurements, thereby enabling the switching between reference and PAS for each measurement step. Consequently, this measurement mode does not allow for simultaneous real-time analysis of PAS and the reference monitor. For this a parallel setup of the two devices would be required.

To conclude the description of the reference device, it is essential to mention that it was also tested for the same cross-sensitivities as the PA signal, in order to verify the reliability of its reference values, since these are not covered by the manufacturer documentation. Water, as the most common variation in ambient air monitoring applications, did not exhibit any interference due to the Nafion tubes installed in the ozone reference device. However, an effect following a linear dependency was observed when varying O_2_ and CO_2_ concentrations over a larger range (i.e. % vol. range), which however is not a realistic change in a practical application. The signal rise with an increasing O_2_ share in the N_2_ buffer gas can be attributed to a weak absorption of the oxygen molecule (shown in Fig. 14 of the appendix) [53]. The decline in signal with the addition of CO_2_ (Fig. 15), on the other hand, cannot be explained by displacement and an associated change in absorption, but a scattering effect of the CO_2_ molecule is conceivable. In contrast to the O_2_ influence, no literature was found to prove this. As both effects demonstrated a linear relation, which is relatively straightforward to compensate for, no further work was conducted to investigate the details of interference in this context. Nonetheless, data-based compensation for these effects is applied in all measurements of this work, including those related to generator cross-sensitivities, as they were previously outlined.

### 2.4 Measurement parameters

Unless otherwise stated, all measurements presented in this chapter adhere to the same measurement conditions and settings to ensure comparability and stringency. The PAS data of each measurement series presented in this work is always offset-corrected by considering both the magnitude and phase of the background signal, which is measured before and after each measurement series, in order to properly apply the digital twin. Thus, the sample gas mass flow is maintained at 500 sccm for all individual measurements, and the PAC is temperature controlled to 30 °C to ensure stable acoustic resonance conditions, also for the non-PAS related measurements. This yields a slight overpressure inside the PAC (total of around 1020 hPa), which results in a resonance frequency of 4704.2 Hz and Q-factor of 89.4 for synthetic air. Before each individual photoacoustic concentration measurement, the exact resonance frequency and quality factor were determined using ARMS. The reference monitor provides data at a rate of 0.25 Hz (with 2 s sample and 2 s background measurement) and each datapoint is averaged over 1 min. The LIA data is read out at a rate of 5 Hz, with a lock-in time constant of 2 s at 18 dB/octave, and is averaged over three datasets, each comprising 100 points each. Consequently, the PAS sensor required 1 minute to obtain a concentration value.

## 3 Results and discussion

### 3.1 O_3_ photodissociation in synthetic air

When determining O_3_ concentrations using PAS, the signal is generated by absorption, i.e. excitation, and subsequent non-radiative relaxation, introducing heat locally within the sample gas. In the UV range, however, the energy of the absorbed radiation is sufficiently high to excite the molecules not only vibronically but also to break bonds, i.e. to photodissociate the analyte with a certain quantum yield Φ. The photodissociation of the analyte can either lead to a decrease in the PA signal as the number of excited molecules decreases [54], exert no influence on the resulting signal [55], [56], or even increase or generate the signal, depending on the dissociation reaction [57], [58]. Thus, the complexity of signal generation may increase based on Φ: For low values, non-radiative relaxation of the originally excited molecule can still dominate. With a high Φ, the reaction products may even be in an excited state, which can contribute significantly to the PA signal through non-radiative relaxation or further exothermic reactions.

**Fig. 3:**
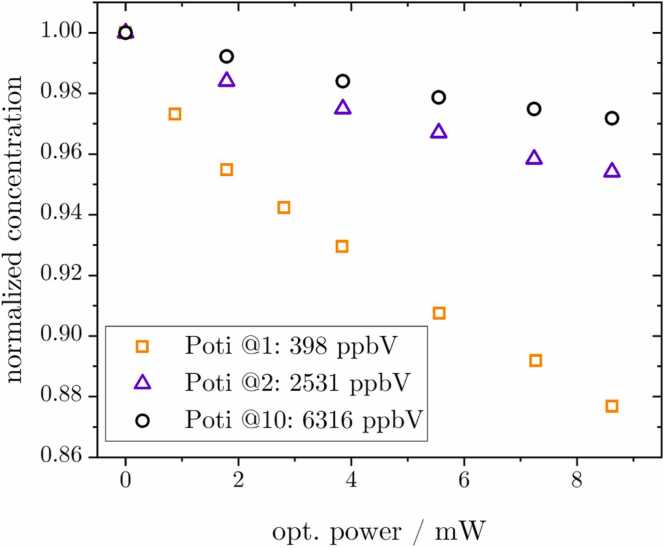
Photodissociation measured with the O_3_ monitor following illumination of the O_3_ gas in synthetic air in the PAC with a 275 nm LED. The stated absolute concentrations of 398 ppbV, 2531 ppbV and 6316 ppbV represent the start value with the LED off. It should be noted that the optical power on the x-axis is only a proportional value, since only a portion of the LED light illuminates the active surface of the power meter.

O_3_ is known to dissociate under UV illumination. Fig. 3 illustrates this as relative decrease in concentration through photodissociation caused by the LED for three different starting O_3_ concentrations. While the negative slope is generally in accordance with the expected behavior, it should be noted that the optical power on the x-axis is not to be taken as an absolute value, since only a part of the LED light hits the active area of the power meter. The dependence of the slope of the relative concentration decrease for different initial concentrations calls for further investigation into photodissociation reaction products.

At the 275 nm emission wavelength, O_3_ is dissociated via two spin-allowed processes. With a quantum yield of 0.9, the excited oxygen atom O(^1^D) with an excited O_2_ molecule (aΔ1g) is formed and with a Φ of 0.1, an oxygen atom and molecule in the ground state O(^3^P) and O_2_ ($X^{3}\sum_{g}^{-}$) are generated [59], [60].

The excited O(^1^D) atom is known to relax in synthetic air to its ground state non-radiatively via collisions with N_2_ [61]. When colliding with O_2_, however, the atom has a certain probability to transfer energy to excite the aΔ1g state and the b∑1g+ state ($k_{\text{total}}=9.8\cdot 10^{8}{\text{ s}}^{-1}{\text{ atm}}^{-1}$) with branching ratios of 0.2 and 0.8, respectively [47]. These two states can subsequently be deexcited via collisions with N_2_, O_2_, H_2_O, or CO_2_ [47] (refer to Table 1 in the appendix for an overview of the respective reaction rates). Overall, this photodissociation experiment provides a theory for understanding the generation of the PA signal in the context of UV O_3_ detection, which is essential for the subsequent cross-sensitivity discussions. Upon reaching O(^3^P), a termolecular reaction, as presented for the O_3_ generator in Eq. (3), with O_2_ and a third collision partner M ($k=3.72\cdot 10^{5}{\text{ s}}^{-1}{\text{ atm}}^{-1}$ [47]) results in the immediate recombination to form O_3_, independent of M [61], [62]. The presented reaction counteracts the effect of photodissociation, thereby resulting in the relative concentration-dependent decrease observed in Fig. 3.

To confirm the theory of recombination in this particular case, the green dotted setup configuration (refer to Fig. 2 for details) is employed using synthetic air. This approach allows for a reduction in flow velocity by splitting the flow, while maintaining a constant level of generated O_3_ concentration and photodissociation. Consequently, the average time required for a molecule or atom to travel from the PAC to the monitor is prolonged, thereby increasing the amount of recombined concentration, and substantiating the theory stated above. An illustration of this measurement is provided in the appendix Fig. 16. A quantitative calculation of the recombination effect in order to model the data shown above was not successful due to the uncertainty in total optical power, i.e. photons interacting with O_3_, as well as missing variables to simulate the consecutively needed collision radii of all gas molecules. Due to the complexity of photodissociation, recombination and acoustic interference of the monitor, an alternating operation of reference and PAS system was chosen.

### 3.2 PAS characterization in synthetic air

**Fig. 4:**
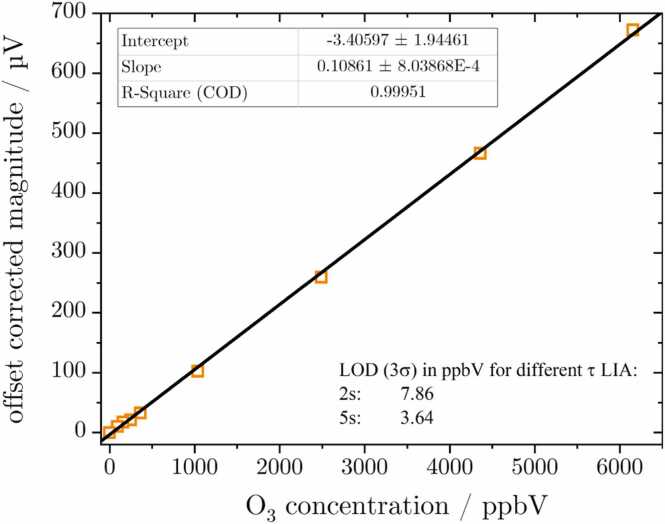
Offset corrected calibration of the UV-LED PAS setup for O_3_ yielding a LOD (3σ) of 7.9 ppbV at a LIA time constant of 2 s with a 3σ noise level of 0.857 µV. A long-term stability measurement with an Allan-Werle deviation analysis can be found in the appendix Fig. 17.

In order to calibrate the PAS measurement system, synthetic air was supplied through the gas system. The O_3_ generator’s potentiometer was utilized to achieve different concentrations, starting from the maximum and progressively decreasing. For each step, the O_3_ monitor was used first to confirm that the concentration was settled and a reference datapoint was recorded. Subsequent to this, the PAS magnitude and phase relative to the LED modulation signal was recorded, followed by the configuration of the next smaller concentration. At the end of the measurement, the background signal is recorded, while the gas is supplied through an O_3_ scrubber. The resulting concentration correlation is corrected for the background signal and displayed in Fig. 4. This is necessary since the background signal at 119.62 µV (-167.9° phase; 3σ noise level of 0.857 µV at τ_LIA_ = 2 s) is rather high compared to the sensitivity of 0.109 µV/ppbV. This is due to the inherently wide emission angle of the LED light, which is difficult to guide perfectly through the cell. Therefore, a significant proportion of the beam hits the PAC walls and generates an offset signal. A long-term stability measurement for an O_3_ concentration can be found in the appendix in Fig. 17. The measured deviation of 0.4 % over a period of 7.5 h indicates a stable measurement system with no instrument specific issued. The alongside plotted Allan-Werle deviation suggests a noise improvement when increasing the averaging time from the 20 s employed in this work up to 200 s. Nevertheless, as shown in the graph in Fig. 4, increasing the LIA time constant is favorable. Additionally, the regular use of a scrubber to check the stability of the background signal is a viable solution to ensure its stability or account for potential drifts.

The limit of detection (LOD) can be calculated for the presented setup using the sensitivity from the calibration in Fig. 4 and the recorded noise level at three times the standard deviation σ. The LOD is thus determined to be 7.9 ppbV with a lock-in time constant of 2 s and 3.6 ppbV for 5 s, respectively. This represents a significant improvement compared to the LODs previously reported in the literature using UV-LEDs for O_3_ detection (LOD (1σ): 1.27 ppmV) [34] and is on par with laser-based setups [32], [33]. The linearity of the PAS signal in response to changes in optical power (according to Eq. (1)) was examined in the course of the calibration measurements photodissociation (visualized in the appendix Fig. 18). This confirms that the well-known linear concentration correlation of the PAS setup is not negatively affected by the photodissociation process involved in generating the PA signal.

### 3.3 Cross-sensitivities

#### 3.3.1 Compensation and model

To investigate the cross-sensitivities of the PA setup in the following subsections, the O_3_ concentration was deliberately set to its highest feasible level. This approach ensures that any effect is clearly observable, well beyond the theoretical detection limits of both the reference and the PAS device.

Since O_3_ and its photodissociation products play a key role in atmospheric chemistry, their interactions with the surrounding molecules, such as deexcitation or reaction processes, have been thoroughly studied. The reaction data summarized in [47] provides an ideal basis for understanding PAS relevant cross-sensitivities and was utilized to establish the rather simple relaxation system depicted in the molecular transition diagram in the appendix in Fig. 19. The associated reaction rates are summarized $k_{1-12}$ in Table 1 in the appendix, respectively. This model was employed in conjunction with the digital twin [36] to address the compensation of cross-sensitivities in the subsequent sections. The digital twin analytically calculates the relaxation cascade based on the input model for a given set of BME sensor and ARMS values as well as a given gas matrix and finally predicts the analyte concentration with respect to the observed magnitude and phase. The refinement factor $C_{\text{corr}}$ applied for this setup was determined to be 6.45 (see Eq. (1)) via averaging over the four measurement series conducted in this chapter. For each measurement, the point for synthetic dry air gas composition at 30 °C was utilized. The factor is likely to be this high as a consequence of balancing out the deviation of the measured optical power value from the real optical power within the PAC resonator and an additional introduction of pressure signal by an increase in the particle number following photodissociation. The averaging yielded a small mean relative deviation for C_corr_ of 0.7 %, which supports the sensor’s signal repeatability during the measurements presented in this work. The concentration values of the DT-compensated data are calculated by solving Eq. (1) for $N_{\text{i}}$, since the other parameters of the equation are only subject to changes in bulk composition and not at trace level. The uncompensated PAS data are calibrated using a standard synthetic air two-point calibration at 30 °C.

Spectral cross-sensitivities towards the studied gases were ruled out through a prior literature review, which did not indicate any interferences.

#### 3.3.2 Cross-sensitivity towards temperature

**Fig. 5:**
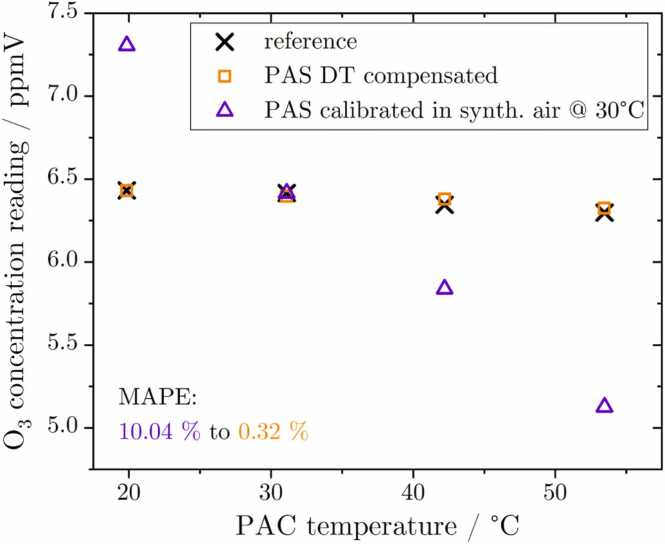
Cross-sensitivity of the PAS setup towards changes in temperature. While the reference shows no significant or functional dependency, the standard calibrated PA signal linearly decreases with rising temperature.

In this experiment, the temperature of the sample gas (synthetic air) in the PAC was altered using the TEC from 20 to 50 °C, with the BME sensor values as gas temperature reference. The O_3_ concentration remained virtually unaffected within this range, as evidenced by the reference measurement data visualized in Fig. 5 (black crosses). On the other hand, the standard calibrated PA amplitude indicated a linear decrease (violet triangles). This phenomenon can be attributed to temperature-dependent acoustic effects, which modify the resonator properties (Q,ω), as measured by the ARMS. In detail, the rise in temperature results in an increase of the resonance frequency due to an accelerated speed of sound. Therefore, the frequency of modulation has been adapted to the change in ω before each measurement step. The decrease in the quality factor is produced by an enhanced energy dissipation caused by increased molecular movement. Additionally, the temperature recorded by the BME sensor within the PAC allows for calculating the parameters γ and $V_{\text{mol}}$, while the temperature dependency of the absorption cross section of O_3_ is negligibly small [63]. Utilizing these variables, the digital twin is capable of efficiently compensating the PA magnitude, considering the dependencies outlined in Eq. (1). Consequently, the compensated PA concentration reading was calculated as illustrated in Fig. 5 (orange rectangles). The mean absolute percentage error (MAPE) of the standard calibrated data compared to the DT compensated improved from 10.04 % to 0.32 %, thereby considerably enhancing the setup performance.

#### 3.3.3 Cross-sensitivity towards O_2_

**Fig. 6:**
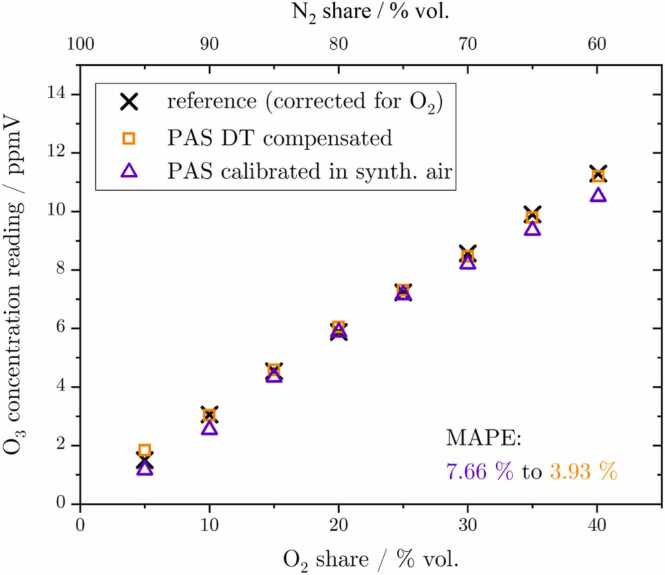
Reference, compensated, and standard synthetic air calibrated PAS signals illustrated for a rising oxygen, i.e. a decreasing nitrogen concentration. The predominant rise in O_3_ concentration is attributed to the dependency of the O_3_ generator on the O_2_ share within the sample.

To investigate the cross-sensitivity of PA O_3_ detection to a change in the O_2_ bulk concentration, N_2_ was successively replaced by O_2_, thus increasing the oxygen concentration within the sample. Generally, both signals are dominated by the change in O_3_ concentration, attributable to the generators’ sensitivity towards varying oxygen exposure (refer to Section 2.3 and Fig. 10 in the appendix). Since the reference monitor shows a cross-sensitivity towards large bulk O_2_ changes as well, as presented by the data in Fig. 14, it has been corrected for its sensitivity and is depicted as black crosses in Fig. 6. On the one hand, the PA setup is again affected by the change in bulk composition, i.e. an alteration of Q, ω and γ, which are considered by the DT in the same way as explained in section 3.3.1. On the other hand, the change in O_2_ concentration influences the non-radiative relaxation path of O(^1^D) and thus the contribution of ϵ to the PA signal. The collision of the excited oxygen atom with N_2_ provides a fast deexcitation reaction ($k_{1}$) to the ground state (^3^P), releasing energy into the system. The presence of O_2_ as a collision partner, however, gives rise to two competing reactions, where in both cases only a part of the energy is released and contributes to the PA signal. This is due to a portion of the energy being transferred to the O_2_ molecule, exciting it to a higher state ($k_{2}$ to aΔ1g and $k_{3}$ to b∑1g+). As already outlined in Section 3.1, these two individual excited singlet states can also release the energy into the gas via collisions with N_2_ and O_2_, however, those transfer processes are relatively slow ($k_{5,6}$ and $k_{9,10}$). Consequently, some part of the energy is no longer available for PA signal generation with respect to the employed frequency of modulation, leading to a loss in PA magnitude. In detail, these competing reactions lead to a decrease in the efficiency of non-radiative relaxation ϵ (with respect to ω) from 0.98 for an O_2_ volume share of 5 % to 0.83 at 40 % vol. oxygen, as calculated by the DT (rates and transitions are summarized in the appendix section Cross-sensitivity relaxation system). Therefore, the standard calibrated PA concentration reading (shown as violet triangles) underestimates the concentration, particularly at higher O_2_ concentrations. In contrast, the DT’s capacity to compensate for the described changes (visualized as orange rectangles) results in an improvement in the MAPE from 7.66 % to 3.93 %. Fig. 6 provides a visual representation of both PA datasets with respect to the O_2_ and N_2_ volume share within the sample.

#### 3.3.4 Cross-sensitivity towards CO_2_

**Fig. 7:**
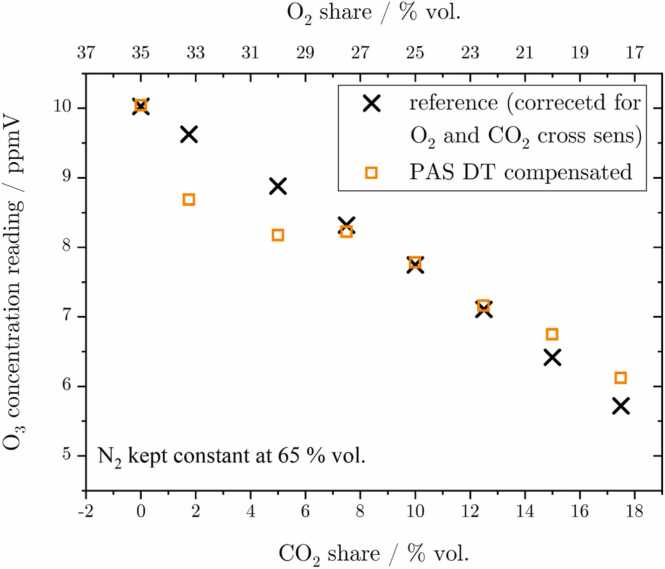
Cross-sensitivity measurement of the PAS signal towards changing CO_2_ concentrations. The reference monitor has to be corrected for its dependency towards O_2_ and CO_2_. The DT data, as demonstrated, offers a means to compensate for the influences introduced by CO_2_, albeit not yet in a fully satisfactory manner. For this reason, a comparison with the standard calibrated PA data was omitted.

The cross-sensitivity of the PAS system towards changes in CO_2_ content was studied starting with a gas mixture containing 35 % vol. O_2_ and 65 % vol. N_2_. Thereafter, the N_2_ share was maintained constant while O_2_ was successively exchanged for CO_2_, with measurements being taken with both the reference and PAS system for every bulk mixture. The predominant drop in both signals once again can be attributed to a decrease in O_3_ concentration due to a reduced generation rate following the O_2_ share. It should be noted that the reference signal visualized in Fig. 7 has been corrected for the monitors’ cross-sensitivities towards O_2_ as well as CO_2_ (see Fig. 14, Fig. 15 for reference). In addition to the change in analyte concentration, the PAS system experiences further attenuation due to acoustic losses caused by the increasing CO_2_ content. This is because the relatively higher molecular mass significantly affects the speed of sound and therefore ω. Additionally, Q and γ exert substantial decrease. After accounting for these effects using ARMS and calculation, relaxational effects have to be considered, too. First, CO_2_ provides a competing reaction with O(^1^D) to the ground state ($k_{4}$), which is faster than the deactivation with N_2_ ($k_{1}$). Second, the deactivation of the excited singlet O_2_ molecule in the aΔ1g state via collision with CO_2_ proceeds too slowly to efficiently contribute to the PA signal ($k_{7}$), while the b∑1g+ state, in contrast, can be efficiently deexcited via $k_{11}$ to the ground state, thereby recovering its energy with respect to PA signal generation at the employed modulation frequency. These two fast reactions result in a steep increase of ϵ once CO_2_ is introduced into the gas. Even with regard to the uncompensated magnitude, this effect counteracts the PA signal attenuation, which is dominated by the decrease in Q. When all the aforementioned effects are accounted for, the DT compensated PAS signal, as shown in Fig. 7, can be obtained. It is evident that the correspondence between the DT compensated signal and the reference values is not yet satisfactory, particularly for the second to third data point. Additionally, the slope of the signal drop appears to be inaccurate, which in combination leads to a significant measurement error. This suggests either an inconsistent measurement, especially for the two outliers, or an incomplete relaxation model. This could indicate either overlooked reactions involving intermediate energy levels that have not yet been considered or a discrepancy in the k values, ultimately resulting in insufficient compensation. However, no further relevant relaxational reactions or competing chemical reactions involving O_3_ and CO_2_ could be found in literature.

#### 3.3.5 Cross-sensitivity towards H_2_O

**Fig. 8:**
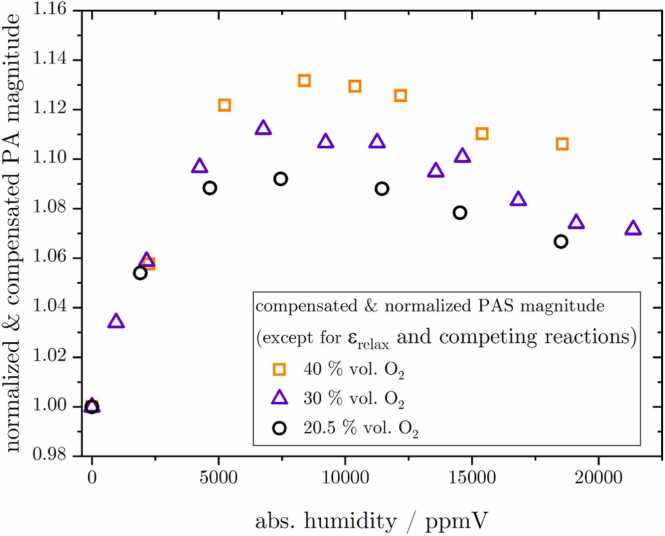
Cross-sensitivity of the PA system towards changes in humidity from 0 to ca. 20 000 ppmV depicted on the x-axis for fixed O_2_ concentrations of 20.5 % vol. (black circles), 30 % vol. (violet triangles) and 40 % vol. (orange squares), utilizing a y-axis representation where the PA magnitude has been compensated for all variables except ϵ and has been normalized to the respective dry measurement point. The resulting graph thus visualizes the change in ϵ maybe blended with competing chemical reactions.

**Fig. 10:**
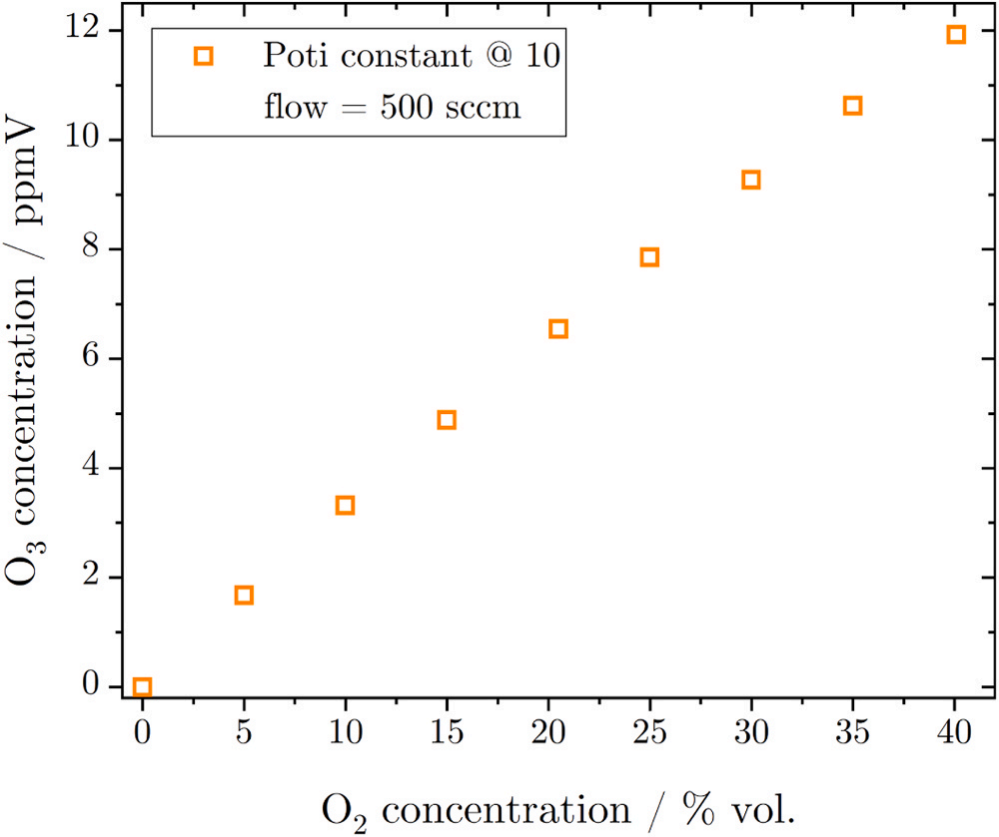
Dependency of the O_3_ generator on O_2_ concentrations in the sample gas at constant potentiometer (10) and flow settings (500 sccm). An increase in the share of O_2_ in the gas directly results in a corresponding rise in generated O_3_ concentration, following (2), (3). The offset signal of the O_3_ monitor due to a change in O_2_ concentration was compensated (see Fig. 14), though its absolute effect is negligible small in this case.

**Fig. 11:**
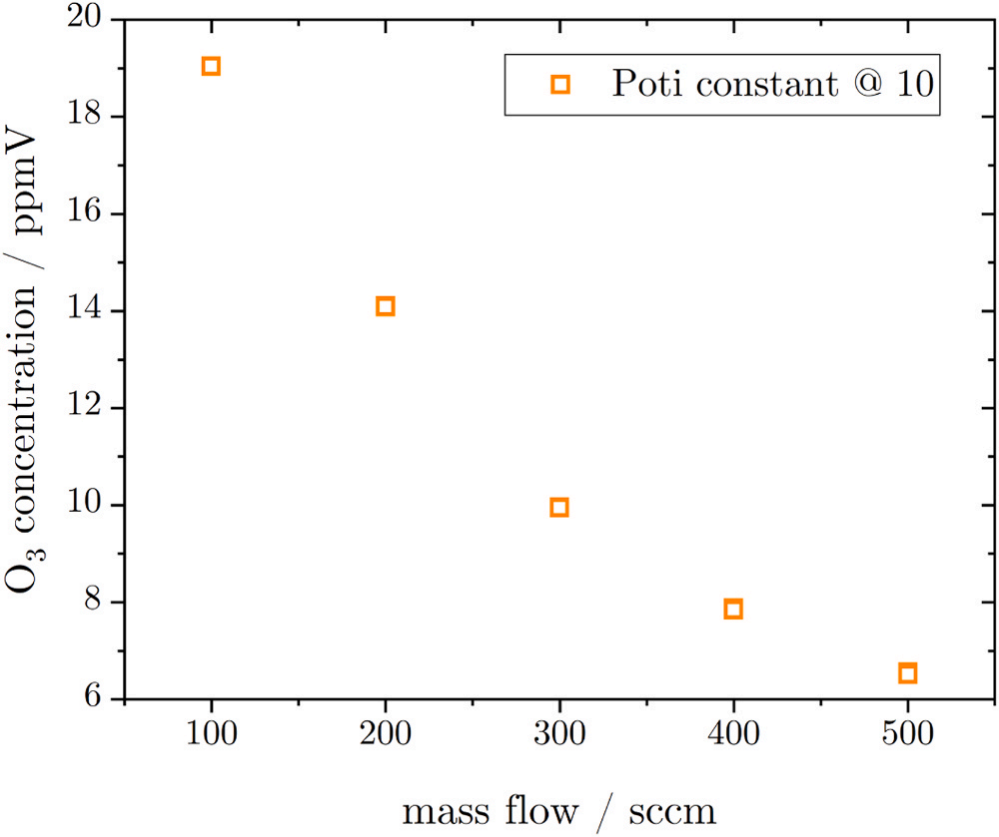
O_3_ monitor concentration readings for a varied mass flow of synthetic air (20.5 % vol.) at a constant potentiometer setting of 10. As the change in flow induced a small (4.6 kPa) change in absolute pressure, the concentration reading was compensated for the individual molar volume. The slow flow velocity associated with a lower mass flow rate results in an increased average residence time of the molecules in the volume irradiated with 185 nm light. This, in turn, leads to an elevated O_3_ generation rate.

**Fig. 12:**
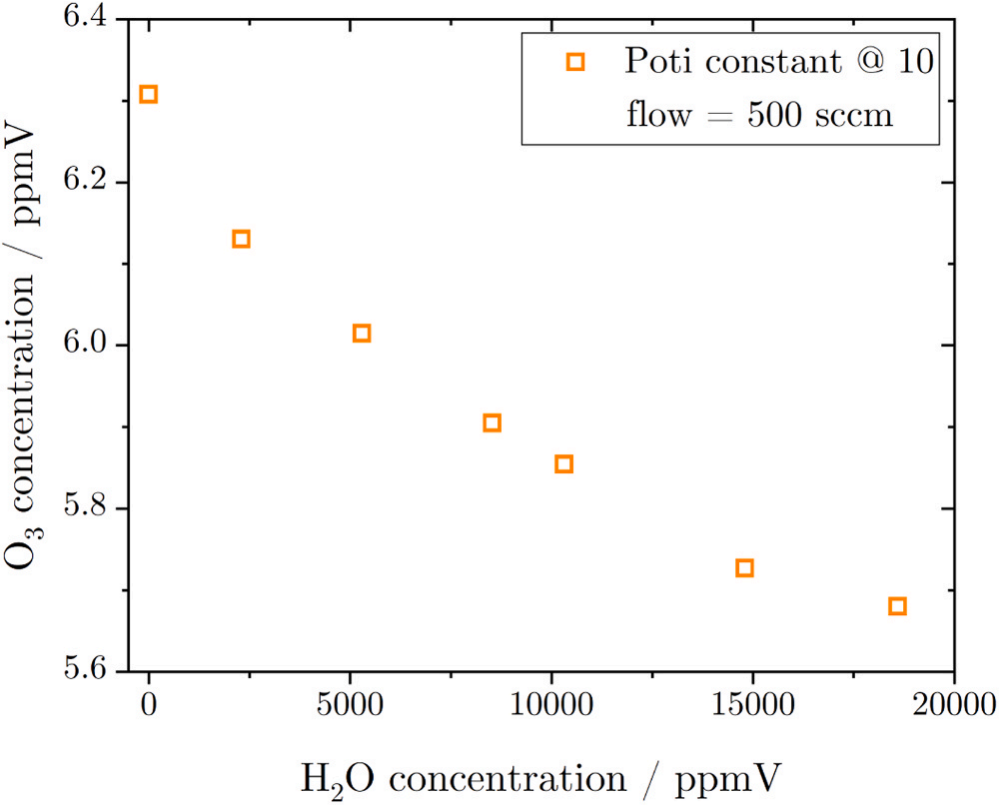
Dependency of the O_3_ generation on the H_2_O share in the synthetic air sample gas. The sample gas was humidified by the setup described in Section 2.3, i.e. humidifying synthetic air prior to its introduction into the O_3_ generator. This encompasses the displacement of a small portion of O_2_ by H_2_O molecules (from 0 to the maximum H_2_O concentration, which implies a decrease in 0.38 % vol. O_2_), that is compensated for using the data from Fig. 10. The remaining decline in concentration can be attributed to the photodissociation of H_2_O and the subsequent reactions, which are comprehensively outlined in the text of Section 2.3.

**Fig. 14:**
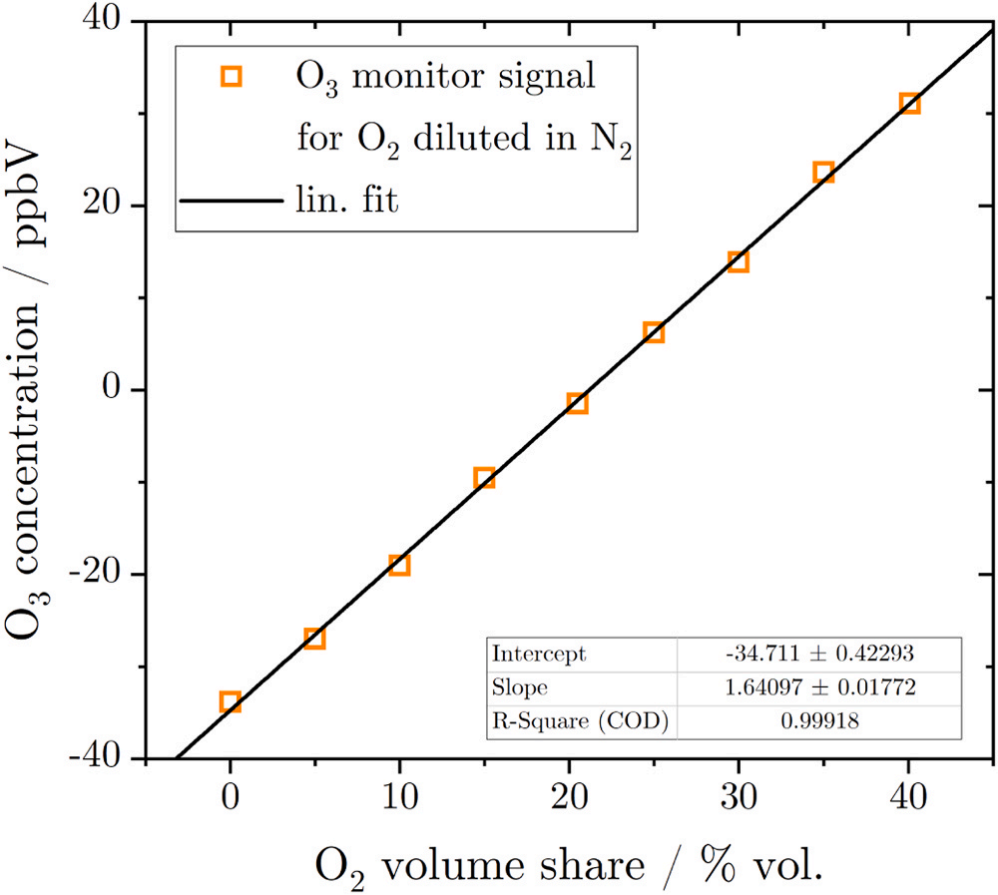
Dependency of the O_3_ monitor concentration signal upon changes in the O_2_ content in N_2_ buffer gas. The observed increase depicted in concentration signal can be attributed to a weak absorption of oxygen at 254 nm that matches the strong absorption of O_3_ with the balancing proportion in concentration shown in this graph.

**Fig. 15:**
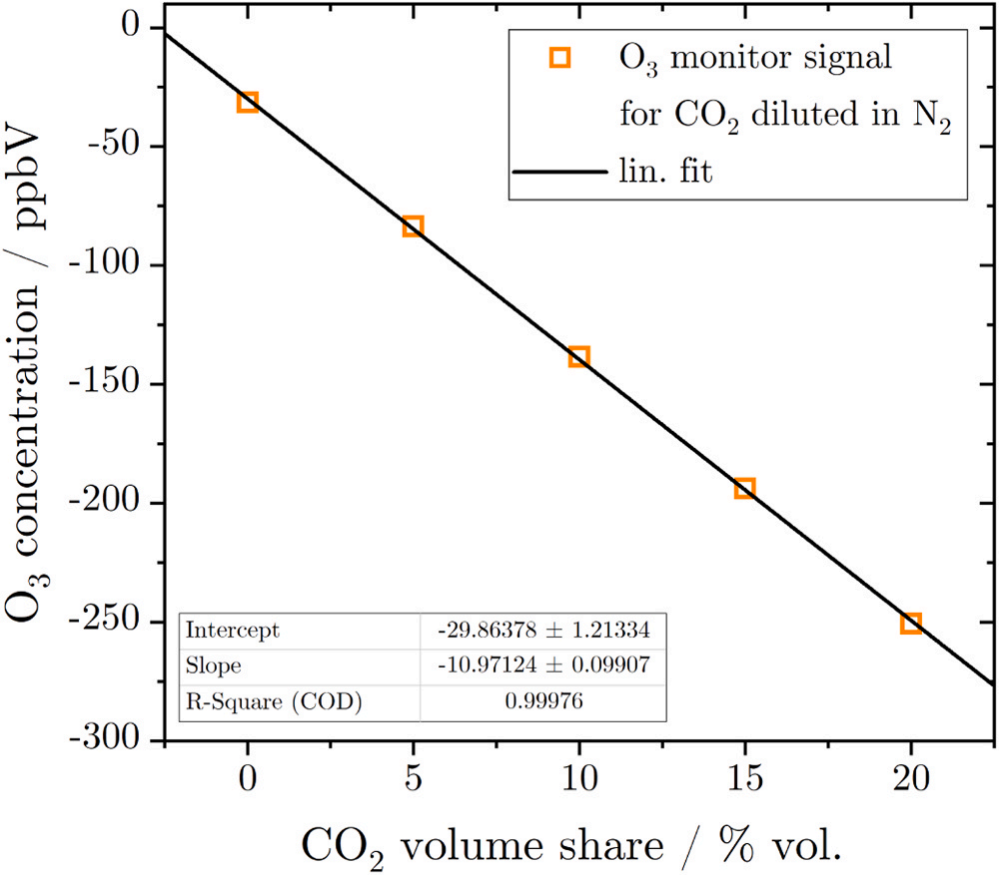
Concentration signal observed with the O3 monitor with the continuous addition of CO_2_, with N_2_ serving as the buffer gas. The decline in signal strength could not be explained by a displacement and absorption feature, given the non-absorbing nature of the N_2_ buffer gas in this wavelength region. However, a scattering effect of the CO_2_ molecule may provide a potential explanation. For a theoretical doubling of CO_2_ levels at ambient conditions, this measured dependency predicts a negligible small accuracy error of −0.439 ppbV, which is lower than the manufacturer stated 2 ppbV accuracy. Although, no further work was conducted in the context of this theory since a data-based compensation with this proportionality was relatively straightforward.

**Fig. 17:**
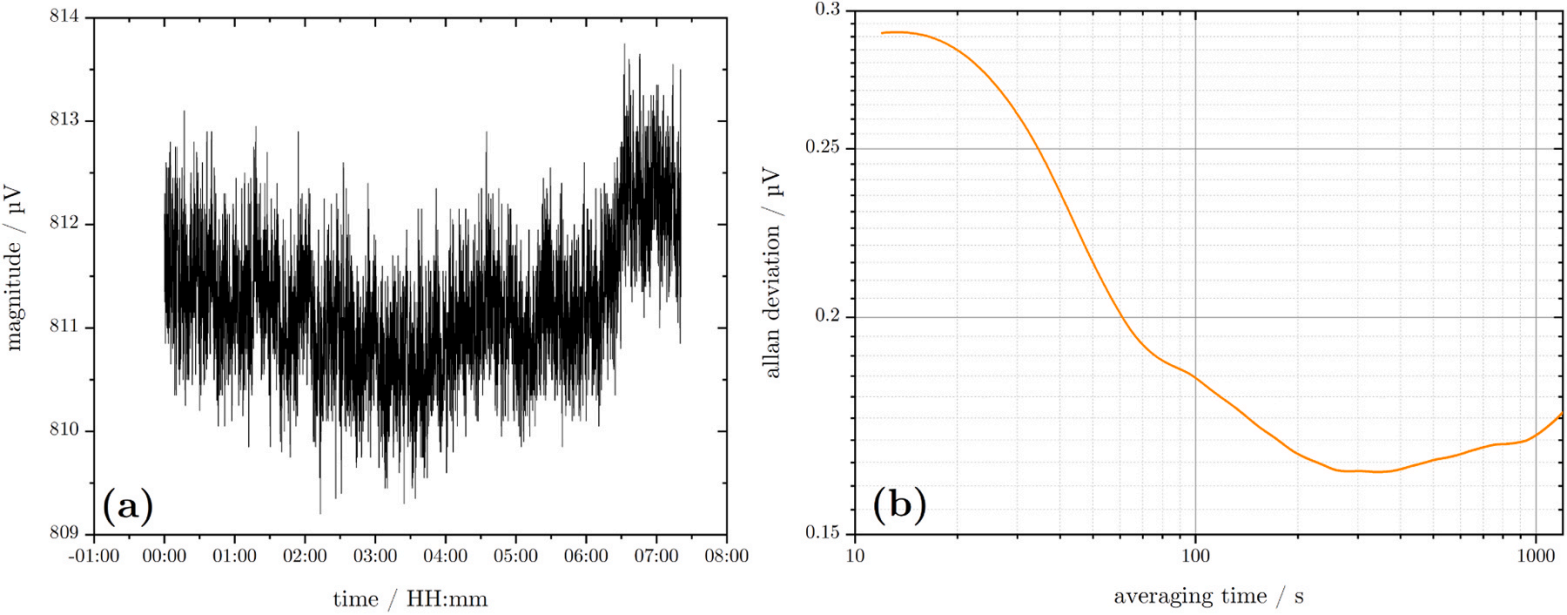
(a) Raw PAS magnitude for a long-term measurement over 7.5 h at an O_3_ concentration of 6.37 ppmV. Within measurement accuracy, the observed deviation of 0.4 % of the concentration indicates no instrument specific issues. However, it should be noted that the reference monitor could not be used for simultaneous verification of stable O_3_ generation. (b) Corresponding Allan-Werle deviation using the raw data from (a), showing a slight noise improvement for longer averaging times beyond 20 s employed in this work, up to 200 s. Nevertheless, increasing the LIA time constant is favorable.

**Fig. 19:**
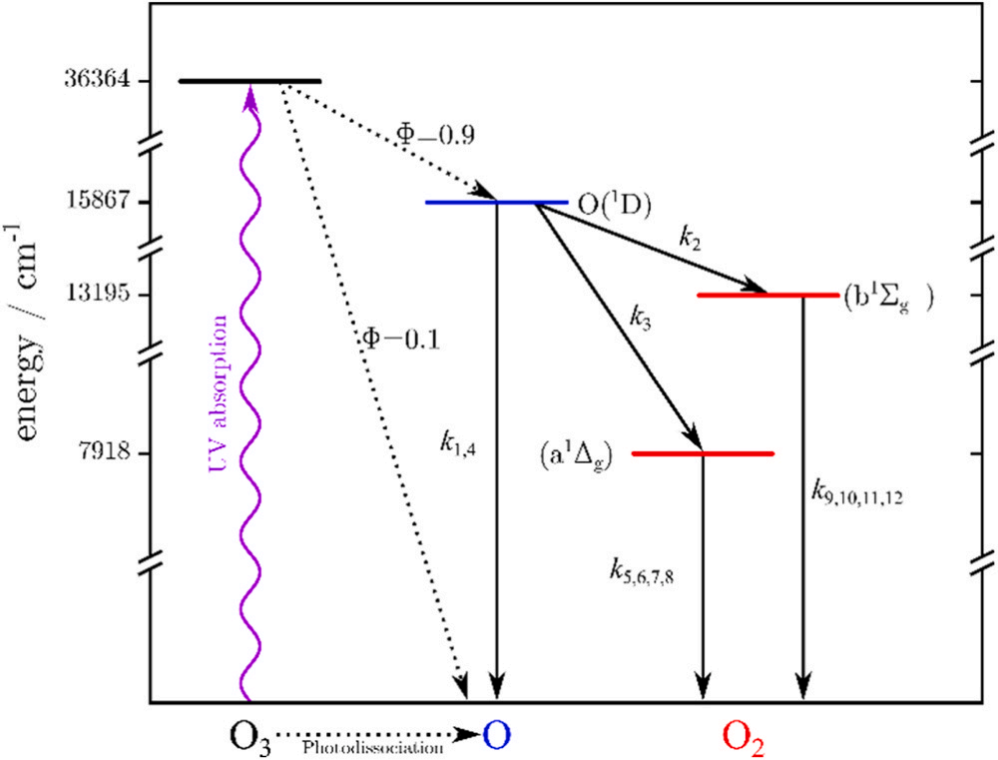
Molecular transition diagram visualizing the deexcitation paths of an excited oxygen atom O(^1^D). This system was employed in conjunction with the respective reaction rates extracted from the summary in [47] in order to establish the DT compensation. The energy level of the O atom was extracted from [67] while those of the singlet O_2_ molecule were gathered from [68].

To investigate the cross-sensitivity of the PA O_3_ signal towards H_2_O, the humidity inside the PAC was adjusted using the system explained in Section 2.3. This involves splitting the flow into a humid and a dry section, controlling the respective flows and thereby the total amount of H_2_O within the sample. The BME sensor readout was used as a reference to measure H_2_O concentration. The initial difficulty in interpreting the PA signal was addressed by conducting the measurement repeatedly for various constant O_2_ concentrations. This approach facilitated a deeper and more comprehensive understanding of the underlying effect. For each humidity level, the PA magnitude was compensated for the measured and calculated changes in Q, ω, γ, $P_{0}$ and $N_{i}$. Then, Eq. (1) was solved for ϵ. Normalizing these values to their respective dry data points yields the curves shown in Fig. 8. As with CO_2_, the H_2_O molecule can deexciting both of the excited O_2_ singlet states via $k_{8}$ and $k_{12}$, however, only the reaction with O_2_(b∑1g+) appears to be sufficiently fast ($k_{12}$) to influence the PA magnitude with regard to the applied modulation frequency. This explains the initial rise that is observed in the graph. These reactions also explain the individual peak values for different O_2_ concentrations, since for higher O_2_ concentrations, more energy is transferred to the oxygen molecule (reactions $k_{3}$ and $k_{4}$, as explained in Section 3.3.3). Consequently, the initial relative signal gain, i.e. rise in ϵ, with increasing H_2_O concentration is higher for larger bulk O_2_ concentrations, as proven by these measurements. This phenomenon was also quantitatively demonstrated by a simulation using the DT. The subsequent or underlying decrease, visible towards higher H_2_O concentrations, however, is assumed to originate from competing reactions. Literature provides two dominant reactions involving O_3_ and H_2_O with an appropriately fast reaction rate [47], [64], [65]

(4) O(^1^D)+H_2_O→2OH

and

(5) O(^1^D)+H_2_O→O_2_+H_2_

Both reactions result in a competing deexcitation of O(^1^D), thereby constantly reducing the PA magnitude, once H_2_O is added to the gas mixture. However, given the exothermic nature of both reactions, it can be hypothesized that a more complex interaction may be accountable for the signal to drop. Since it is not possible to directly monitor the O(^1^D) concentration with the current setup, no reliable evidence was obtained to support this assumption. Furthermore, the DT is not yet capable of accounting for chemical reactions at a given rate where the products differ from the reactants and either energy is released or consumed. As a result, this cross-sensitivity could not yet be fully modeled and compensated for.

Finally, the presented PAS sensor presents a promising low-cost UV LED-based trace gas measurement system for O_3_ monitoring. With a LOD of 7.9 ppbV, it meets the necessary requirements for potential application in industrial safety processes. For environmental monitoring, however, the remaining challenges need to be addressed, and for both applications real-world samples must be studied. In particular, high dynamic range applications could benefit from the PAS system, as direct absorption-based spectrometers are limited in their useable detector range at higher O_3_ concentrations. For further enhancement of sensitivity and detection limits of the PAS system, various approaches are outlined in [66].

## 4 Conclusion

We presented a PAS-based O_3_ detection system, utilizing low-cost components such as a MEMS microphone as detector and a UV-LED as light source. For the first time, the PAS signal origin as a consequence of photodissociation of ozone, induced by UV absorption is introduced. Besides a general increase in particle density, photodissociation yields excited oxygen atoms O(^1^D), which subsequently relax non-radiatively, thereby contributing to the PA signal. Based on that, our PA setup achieves a remarkable LOD (3σ) of 7.9 ppbV, providing a significant enhancement to previously reported LED-based setups. This sensitivity and large dynamic range, along with the use of low-cost components, underscore the potential of this UV-LED PAS setup with a technology readiness level in between 3 and 4.

Additionally, this study highlights the importance of identifying the various contributions to and complex pathways of PA signal origin in the UV range. Without knowledge of the underlying processes, it is hardly feasible to fully understand and ultimately compensate for the cross-influences. Here, we presented the effects of temperature and changing O_2_ concentrations and introduced a compensation model based on a previously published digital twin. The interactions of O(^1^D) and the first two excited singlet O_2_ states within the bulk composition have been identified to cause the observed cross-sensitivities. However, the results from the experiments with changes in the bulk composition regarding CO_2_ and H_2_O still leave room for improvements in the compensation model.

## Funding

This work has received essential financial support through the BreathSens project, funded by the German Ministry of Education and Research (BMBF) under grant code 13GW0325C, and the PreSEDA project, funded by the German Federal Ministry for Economics and Climate Action (BMWK) under grant code 03EN2028A. The publication fee was covered through funding provided by the DEAL project.

## CRediT authorship contribution statement

**Matysik Frank-Michael:** Writing – review & editing, Validation, Supervision, Methodology, Conceptualization. **Bierl Rudolf:** Writing – review & editing, Supervision, Project administration, Funding acquisition. **Pangerl Jonas:** Writing – review & editing, Validation, Methodology. **Jobst Simon:** Writing – review & editing, Validation, Software, Methodology. **Rück Thomas:** Writing – review & editing, Validation, Project administration, Methodology, Investigation, Conceptualization. **Escher Lukas:** Writing – review & editing, Writing – original draft, Visualization, Validation, Software, Methodology, Investigation, Data curation, Conceptualization.

## Declaration of Competing Interest

The authors declare that they have no known competing financial interests or personal relationships that could have appeared to influence the work reported in this paper.

## Figures and Tables

**Fig. 9:**
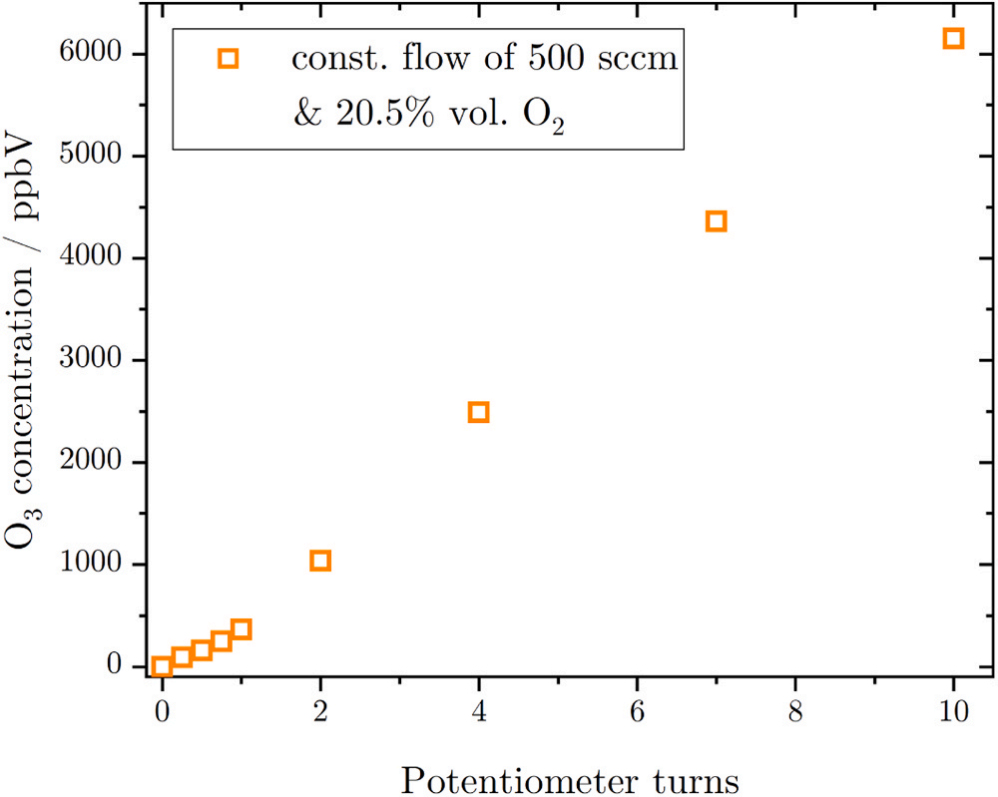
Correlation between the potentiometer turns on the AOS ozone generator and the concentration of O_3_ measured by the reference monitor at a constant flow rate of 500 sccm and 20.5 % vol. O_2_. A higher number of potentiometer turns results in an increased 185 nm light output power, i.e. higher O_3_ generation rate. Given the observation of hysteresis in the potentiometer concentration setting, an attempt was made to maintain its value at a constant level throughout a single measurement series.

**Fig. 13:**
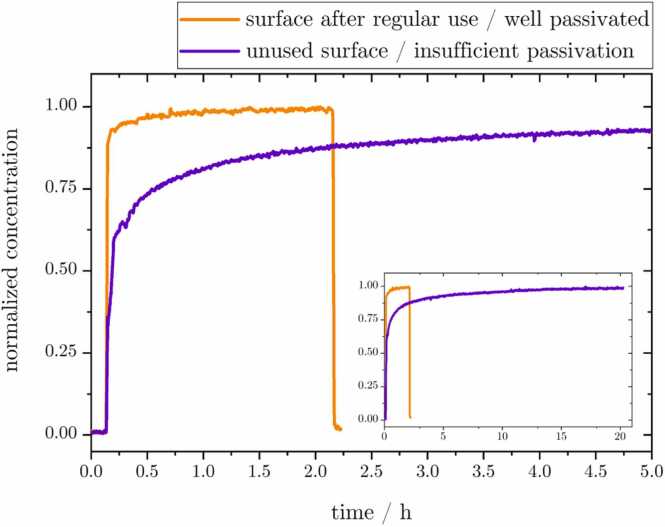
Comparison of the settling time of an unused PAC and gas setup with inadequate passivation and a regularly used and well-passivated gas setup. Both curves were set with the potentiometer to reach a concentration of 400 ppbV O_3_ and are normalized to their respective maximum. A notable decrease in settling time occurs as consequence of the passivation, which has resulted in a diminished activity level at the surface of the PAC. This ultimately leads to a reduction of the time required for measurements in subsequent experiments within this study.

**Fig. 16:**
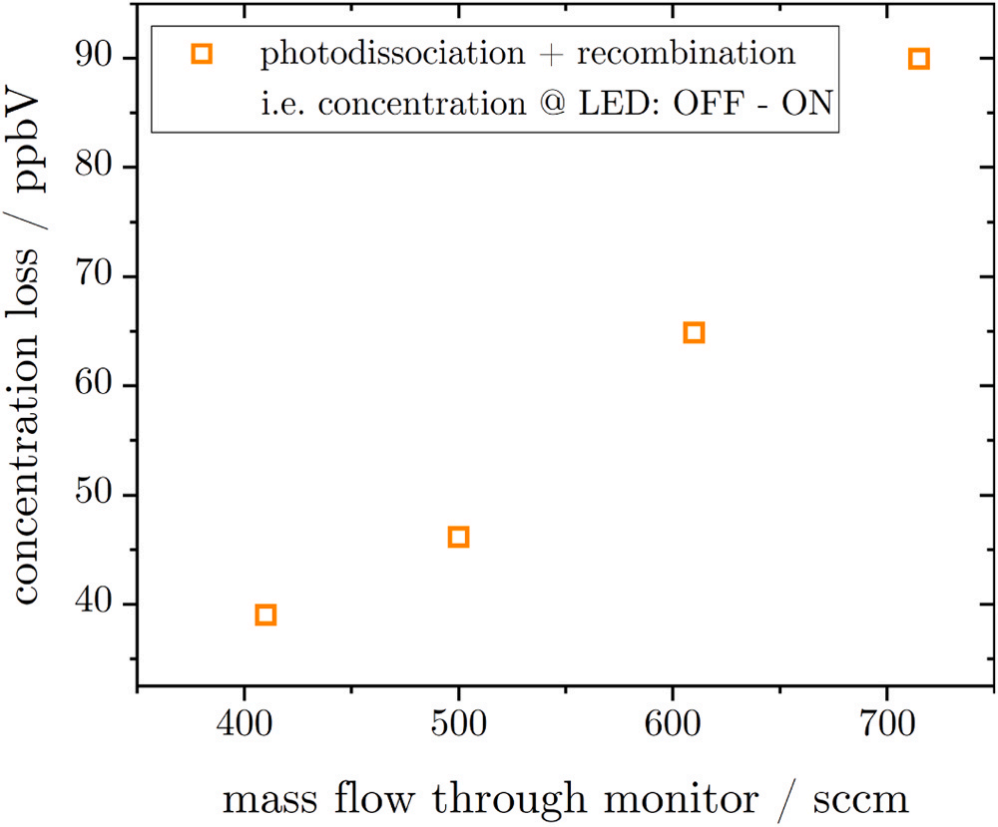
O_3_ concentration measurement with the reference monitor for different mass flows, i.e. changing the flow velocity and thus travel times for molecules and reaction or photodissociation products from the PAC to the reference. For each flow, the photodissociation effect in combination with the occurring recombination is measured. The decreasing concentration loss indicates a stronger recombination for longer travelling times, which confirms the theory stated in Section 3.1.

**Fig. 18:**
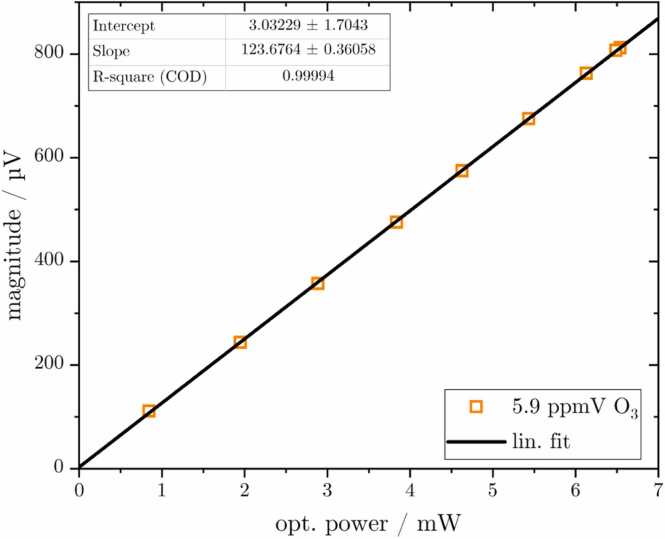
Correlation between the PA signal and a change in optical power. This finding suggests that the signal generation remains undisturbed by the photochemical processes outlined in chapter 3, as evidenced by the observed linear behavior that follows Eq. (1).

**Table 1:** Summary of the reactions and their respective rate constants used within the DT for simulation of the PA signal with regards to cross-sensitivities.

	**Reaction**	$\frac{∆E}{hc_{0}}$**in cm**^**−1**^	**Reaction rate**k**in s**^**−1**^**atm**^**−1**^
$\left(1\right)$	OD1+M→O(P3)+M	−15867	$\begin{array}{c} {k_{1}^{N_{2}}}^{\;}=7.686\cdot 10^{8}\;\\ {k_{4}^{{\mathrm{CO}}_{2}}}^{\;}=1.859\cdot 10^{9} \end{array}$
$\left(2\right)$	OD1+O2→O(P3)+O2b∑1g+	−2672	$k_{2}^{O_{2}}=7.835\cdot 10^{8}$
$\left(3\right)$	OD1+O2→O(P3)+O2aΔ1g	−7949	$k_{3}^{O_{2}}\;=1.959\cdot 10^{8}$
$\left(4\right)$	O2aΔ1g+M→O2+M	−7918	$\begin{array}{c} k_{5}^{O_{2}}=4.21\cdot 10^{1}\;\\ {k_{6}^{N_{2}}}^{\;}=2.48\cdot 10^{-1}\;\\ {k_{7}^{{\mathrm{CO}}_{2}}}^{\;}=4.96\cdot 10^{-1}\;\\ {k_{8}^{H_{2}O}}^{\;}=1.19\cdot 10^{2} \end{array}$
$\left(5\right)$	O2b∑1g++M→O2+M	−13195	$\begin{array}{c} k_{9}^{O_{2}}=9.67\cdot 10^{2}\;\\ {k_{10}^{N_{2}}}^{\;}=5.21\cdot 10^{4}\;\\ k_{11}^{{\mathrm{CO}}_{2}}=1.04\cdot 10^{7}\;\\ k_{12}^{H_{2}O}=1.46\cdot 10^{8} \end{array}$

## Data Availability

Data will be made available on request.

## Appendix

The figures presented in this chapter serve to illustrate the main section of this paper, since they do not constitute a grave addition to the presented theses and arguments, but rather visualizing aspect. They are therefore included here as supplementary material for the sake of improved clarity. Unless otherwise stated, all reference monitor data points are collected for a sample gas mass flow of 500 sccm using synthetic air and each datapoint is averaged over 1 minute at a data rate (device limited) of 0.25 Hz.
